# Perineural hematoma following lumbar injection presenting as a neurosurgical emergency

**DOI:** 10.31744/einstein_journal/2025RC1483

**Published:** 2025-10-13

**Authors:** Guilherme Pianowski Pajanoti, Matheus Neves Castanheira, Delio Eulalio Martins, Michel Kanas, Marcelo Wajchenberg, Nelson Astur

**Affiliations:** 1 Hospital Israelita Albert Einstein São Paulo SP Brazil Hospital Israelita Albert Einstein, São Paulo, SP, Brazil.; 2 Instituto Cohen Ortopedia São Paulo SP Brazil Instituto Cohen Ortopedia, São Paulo, SP, Brazil.

**Keywords:** Perineural, Hematoma, Injections, Low back pain, Sciatica, Radiculopathy, Descompression, surgical, Magnetic resonance imaging

## Abstract

Lower back pain and sciatica account for approximately 40% of work-related absences, with management options ranging from conservative measures, such as rest and analgesia, to surgical intervention. Lumbar epidural steroid injections and facet joint blocks are frequently used for both diagnostic and therapeutic purposes. While most complications are minor (2.4%-9.6%), severe events, including infection, hematoma formation, and spinal cord infarction, have been reported. This case presents a perineural hematoma manifesting as acute radiculopathy, necessitating urgent surgical decompression. The patient was a 55-year-old woman with a 7-month history of low back pain radiating to her right leg, unresponsive to conservative treatments, who subsequently underwent facet and nerve root injections. Magnetic resonance imaging demonstrated lumbar degeneration with Modic changes and multilevel disc bulging. The procedure, performed under fluoroscopic guidance with contrast and therapeutic agents, was initially uneventful. However, on the third day post-injection, she developed acute left leg weakness and sensory impairment. Repeat magnetic resonance imaging showed an abnormal signal in the left L3-L4 foramen compressing the L3 nerve root, raising suspicion for a perineural hematoma. Urgent surgical decompression was performed, and pathological examination confirmed organizing hemorrhage. The patient's neurological symptoms improved rapidly, with complete recovery achieved within a month. The literature indicates that hematomas may arise from increased epidural pressure or direct needle injury, even in the absence of signs of bleeding. Practitioners should remain vigilant for post-injection hematomas, as delayed recognition can result in permanent neurological deficits. Magnetic resonance imaging is essential for timely diagnosis, and urgent decompression may optimize outcomes, given that earlier intervention is associated with better recovery.

## INTRODUCTION

Low back pain and sciatica are common pain conditions, accounting for approximately 40% of work-related absences.^(1)^ Treatment options range from rest, analgesia, physical therapy, and exercise to percutaneous interventional procedures and spinal surgery.^(2)^ Lumbar epidural steroid injections (ESI) for radicular pain and lumbar facet blocks for axial back pain are increasingly performed for both diagnostic and therapeutic purposes.^(3)^ The use of spinal injections has grown significantly over the past few decades, with an annual increase of 5-6% between 2000 and 2014 in the USA.^(4)^ Steroids exert therapeutic effects through their anti-inflammatory and nociceptive-stabilizing properties.^(5)^

Pain associated with herniated discs arises not only from mechanical compression but also from chemical irritation of the nerve root, attributable to high concentrations of nociceptors and inflammatory mediators in the perineural environment.^(6)^ Additionally, medial branch blocks using local anesthetics, with or without steroids, have demonstrated therapeutic benefits for lumbar facet joint pain,^(7)^ receiving strong recommendations for short- and long-term pain relief in the management of chronic facet joint pain.^(7)^

Corticosteroids are believed to reduce the inflammatory component of nerve root pathology.^(7,8)^ While the precise mechanism of action in radicular pain is not fully understood, sciatica is known to result from the ectopic firing of chemical mediators, including prostaglandin E and other inflammatory molecules, along with mechanical nerve root compression.^(9,10)^

Fluoroscopic guidance allows precise needle placement in the ventrolateral epidural space, where most pain generators are located, thereby achieving superior pain relief.^(11)^ However, despite fluoroscopic guidance is frequently performed in surgical settings, lumbar injections are not without risks.^(12)^

Most complications are classified as "minor," with an incidence of 2.4%-9.6%.^(12)^ These include vascular penetration, nonpositional headaches, back pain, worsening leg pain, facial flushing, transient nerve root irritation, and vasovagal reactions. Rare but severe complications, such as infection, hematoma, intravascular injection of medication, spinal cord infarction, gas embolism, and hypersensitivity reactions, have also been reported.^(12-14)^ Here, we present a case of perineural hematoma occurring three days after a facet and foraminal block, compressing the left L3 nerve root and resulting in acute lumbar radiculopathy with motor deficits that required urgent surgical lumbar decompression.

## CASE REPORT

**Figure 1:**
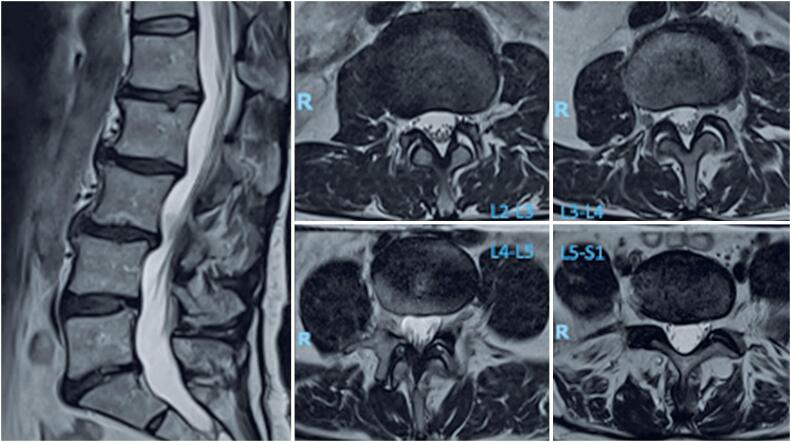
Sagittal central cut and axial cuts by level, T2-weighted magnetic resonance imaging pre-procedure

A 55-year-old woman presented with a seven-month history of low back pain radiating to the right thigh and leg, unresponsive to analgesia and motor physical therapy. The Visual Analog Scale (VAS) recorded lumbar pain as 6/10 and leg pain as 7/10. Initial physical examination revealed normal findings, with intact lower limb strength, no sensory deficits, normoreflexia, and negative provocation tests. Magnetic resonance imaging revealed a scoliotic posture of the lumbar spine with minimal degenerative retrolisthesis from L2 to L4, diffuse disc degeneration (most pronounced at L4-L5 and L5-S1), Modic type I changes, bilateral disc bulging at L3-L4, L4-L5, and L5-S1 (right greater than left), and multilevel facet hypertrophy (Figure 1).

**Figure 2:**
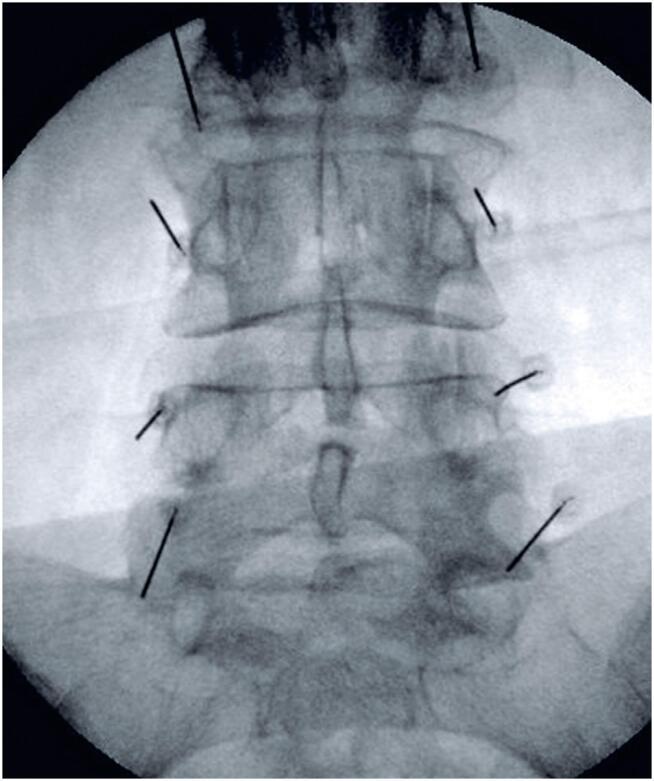
Spinal needle positioning for facet infiltration with chemical denervation of the medial dorsal branch under fluoroscopic guidance

**Figure 3:**
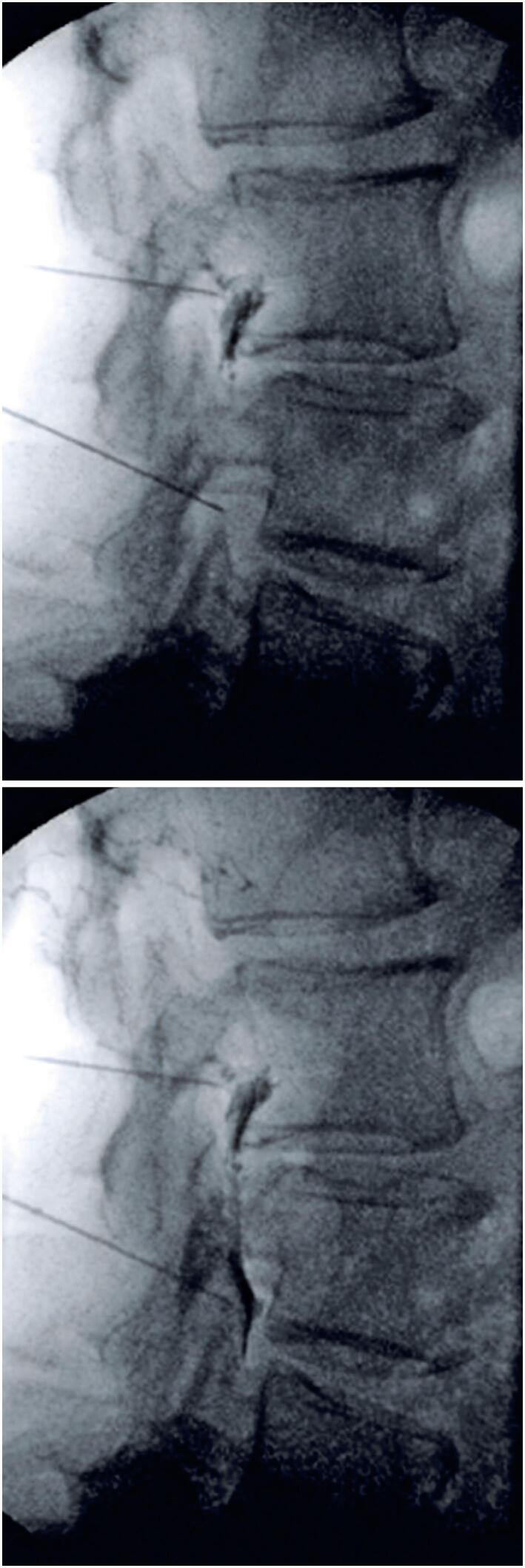
Spinal needle positioning for L3-L4 and L4-L5 foraminal infiltration for ESI with staining of L4 and L5 roots using Omnipaque contrast

Given persistent pain despite physiotherapy, bilateral lumbar facet injections from L2 to S1 and right L3 and L4 nerve root injections were performed under fluoroscopic guidance. Notably, the patient had no known history of coagulopathy, liver disease, anticoagulant therapy, or nonsteroidal anti-inflammatory drug use at the time of diagnosis and intervention. Preoperative laboratory showed an INR of 1.0, platelet count of 270,000, and fibrinogen level of 326. The medial branch dorsal facet block technique was employed (Figure 2), along with a standardized transforaminal approach under fluoroscopic guidance. During the foraminal injection, the needle was positioned without any blood or cerebrospinal fluid return, and no paresthesia was induced during needle placement. Omnipaque contrast agent (0.5mL) was injected under fluoroscopic guidance (Figure 3). A 5mL solution containing 1.5mL of triamcinolone (20mg/mL), 1.5mL ropivacaine (7.5mg/mL), and 2mL 0.9% saline was administered in fractional doses. The procedure was completed without complications, and the patient remained asymptomatic in the recovery room with no new signs or symptoms of neural compression, such as recent-onset numbness, weakness, bowel or bladder dysfunction, or severe radicular back pain.

**Figure 4:**
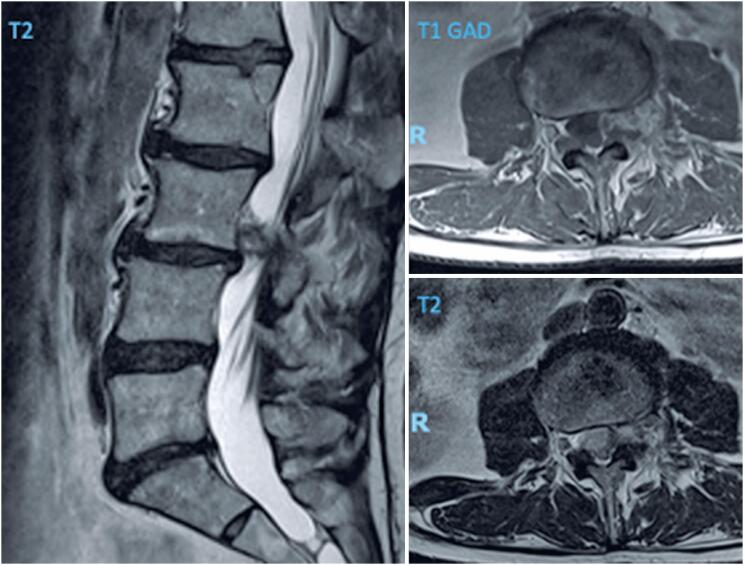
Sagittal central cut and axial images of the L3-L4 level from the post-procedure magnetic resonance imaging

On the third day post-injection, the patient returned to the clinic with progressive weakness in the left lower limb, sensory loss in the left thigh, and gait dysfunction. Visual Analog Scale lumbar pain was 4/10, leg pain was 9/10, and the Oswestry Disability Index (ODI) indicated moderate disability (40%). Physical examination revealed grade 4 strength in the left quadriceps, sensory loss in the L3 dermatome extending from the anterolateral thigh to the anteromedial left leg, and a positive Nachlas test result on the left side. The patient was referred for urgent gadolinium-enhanced MRI, which showed an abnormal focal signal in the left L3-L4 foraminal region, extending from the lateral recess to the extraforaminal region, compressing the left L3 nerve root anteriorly, with peripheral inflammatory enhancement (Figure 4).

**Figure 5:**
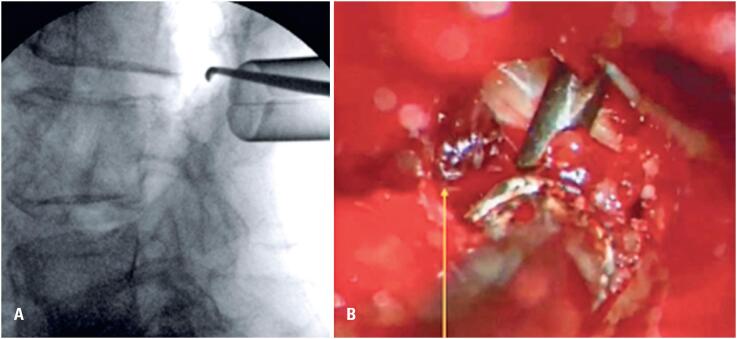
A and B. Fluoroscopic image of the tubular decompression approach with a marker at the left L3-L4 foraminal region, and intraoperative microscopic image showing the perineural hematoma (yellow arrow)

Infection and extruded disc herniation were discussed with the radiologist as possible etiological factors, but were considered unlikely based on the imaging findings and the patient's symptom onset, leading to perineural hematoma as the primary diagnostic hypothesis. The patient was admitted for urgent surgical treatment, which involved left L3-L4 tubular lumbar decompression (Figures 5A and 5B).

**Figure 6:**
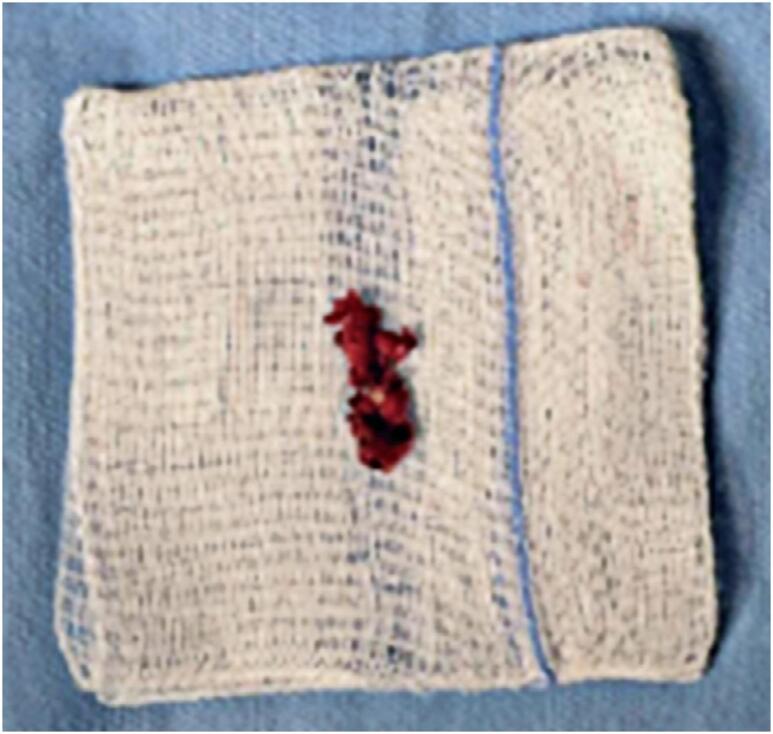
Pathological examination showing macroscopic images of the hematoma and fibroconnective tissue removed during surgical decompression

Histopathological examination confirmed the diagnosis of a hematoma with small fragments of fibroconnective tissue, associated with areas of organizing hemorrhage (Figure 6).

On the first postoperative day, the patient's symptoms began to improve, with full recovery to baseline within one month, a VAS leg pain of 1/10, and an ODI indicating minimal disability (10%). Strength, sensation, and pain relief were restored to preprocedural levels.

The study was approved by the Ethics Committee of *Hospital Israelita Albert Einstein*, CAAE: 82467924.5.0000.0071; # 7.062.651.

## DISCUSSION

Lumbar injections are associated with various known side effects, including infections, hematomas, intravascular injections, neural trauma, dural puncture, and gas embolism.^(12)^ One potential cause of epidural hematoma is vessel injury near the foramen, resulting from direct needle trauma.^(12)^ Such vascular injury increases the likelihood of intravascular administration of medications, which can trigger bleeding and hematoma formation. The risk is heightened in patients on anticoagulants or those with inherited or acquired coagulation disorders, but can also occur in patients without such histories, as seen in this case, necessitating extreme caution during the procedure.^(15)^

To gain a clearer understanding of the location, potential etiology, and consequences of epidural hematomas, recognizing the anatomical relationships within the spinal column is crucial. Epidural hematomas typically form dorsally or posterolaterally to the dural sac, as the dural sac tightly adheres to the posterior longitudinal ligament on the ventral aspect of the spinal canal.^(16)^ The epidural space is bordered posteriorly by the ligamentum flavum and periosteum of the laminae, and laterally by the pedicles of the vertebrae and intervertebral foramina. This space is filled with fat, areolar tissue, lymphatic vessels, arteries, and a highly vascular venous plexus. The intervertebral foramina contain the spinal nerve roots, dorsal root ganglia, spinal arteries, and communicating veins.^(15)^

One vascular structure that may be injured during foraminal lumbar injections is the internal vertebral venous plexus (Batson's plexus), which surrounds the spinal canal.^(17)^ The anterior portion of the internal vertebral venous plexus, composed of two longitudinal veins, covers the floor of the vertebral canal and lies on the posterior surface of the vertebral bodies and the intervertebral disc, near the target area for transforaminal injections. This plexus also includes two longitudinal posterior veins that line the roof of the vertebral canal, situated posterolaterally within the spinal canal.^(18)^ At each intervertebral foramen, the two internal vertebral venous plexuses communicate with the ascending lumbar veins, which run anteriorly to the base of the transverse process. The plexus lacks valves and consists of thin-walled veins that can distend to large calibers. Venous accumulation may gradually develop over several days, with symptoms emerging once the hematoma reaches a critical volume, compressing the spinal nerve roots and causing neural ischemia.^(18)^

The radicular artery, which runs along the spinal nerve root, can also be damaged by direct needle trauma. Radicular arteries arise from the lumbar arteries, which originate from the aortic branches, and supply blood to the spinal nerves and nerve roots.^(17)^ Two venous systems are involved in the drainage of the lumbar spinal nerve roots: the distal veins drain into the lumbar vein at the foramen level, while the proximal radicular veins drain into the venous plexus of the spinal cord.^(19)^

In addition to direct needle injury, hematoma formation may also result from vessel damage caused by increased pressure within the epidural space. Kim et al. reported a case involving an 82-year-old man who underwent a right L2-L3 foraminal lumbar injection for lumbar spinal stenosis.^(20)^ The following morning, the patient presented with worsening back pain and motor deficits. Magnetic resonance imaging revealed fluid accumulation in the posterior epidural space, extending from T11 to L1. Surgical decompression of the epidural hematoma was performed on the same night, involving bilateral laminectomy from T12 to L2, and the coagulated hematoma mass between T12 and L1 was excised. During surgery, continuous bleeding was observed from the posterior surface of the T12 vertebral body, which was identified as the source of the epidural hematoma. The patient had a favorable recovery, with resolution of motor deficits and back pain within two weeks post-surgery.^(20)^

Another case reported by Shanthanna et al.^(21)^ describes an epidural hematoma occurring at the T10-T12 level following an epidural block at L3-L4. The authors suggested that vessel rupture caused by excessive drug injection pressure could explain why the epidural hematoma developed distant from the injection site.^(21)^ In patients with spinal stenosis, a narrowed spinal canal increases epidural pressure. If additional pressure is applied during drug injection, it can exceed the structural integrity of the venous plexus vessel wall, leading to hematoma formation.^(21)^ Other reported cases support this hypothesis, indicating that hematomas may develop as a result of similar mechanisms.

Gungor et al.^(22)^ reported a contralateral epidural hematoma following an injection, while Nan et al.^(23)^ described two cases of epidural hematoma following lumbar facet injections. In the first case, a 60-year-old man presented with bilateral buttock pain radiating to the right thigh five days after undergoing acupuncture and a facet joint block. Magnetic resonance imaging revealed an epidural mass in the lumbosacral region, which compressed the thecal sac posteriorly. This finding led to a laminectomy at L4, L5, and S1, and the removal of the epidural hematoma. However, no source of bleeding was identified during the intraoperative examination.^(23)^ The patient recovered completely and was discharged two weeks postoperatively.

In the second case, a 41-year-old woman developed severe low back pain radiating to the right leg four days after facet joint blocks. Magnetic resonance imaging revealed an epidural mass extending from L4 to L5, which compressed the thecal sac. Although laminectomy was initially planned, her clinical symptoms progressively improved, leading to a conservative approach with close observation. After one month, the patient made a full recovery of motor and sensory functions without requiring surgical intervention.^(23)^

In the present case, the hematoma likely resulted from increased pressure within the epidural space, possibly due to a contralateral foraminal injection or direct needle trauma during the facet injection at the hematoma level. When the needle entered the right L3-L4 intervertebral foramen, the epidural hematoma was located in the contralateral foraminal and extraforaminal regions. Facet injections at the same level and on the same side may have contributed to the formation of the hematoma.

Physicians performing such procedures must remain acutely vigilant for epidural hematomas, as these can result in permanent and debilitating damage.^(24)^ In addition to direct vessel injury, epidural or perineural hematomas can also occur as a consequence of increased pressure during drug injection, even in the absence of visible bleeding or the use of anticoagulants. In this case, nerve ischemia resulting from the hematoma presented clinically as left quadriceps weakness, sensory loss in the left thigh, and a positive Nachlas test. However, other neurological deficits, including bowel and bladder dysfunction, could arise depending on the location and size of the hematoma.

Epidural hematomas should be promptly evaluated using MRI, as several case reports and studies underscore the necessity for immediate surgical decompression. The inclusion of VAS assessments in this case provides valuable insight into the dynamic nature of post-injection and post-surgical decompression pain perception. The significant increase in leg pain by the third day post-injection, which corresponded with the onset of neurological deficits, reinforces the hypothesis of an evolving hematoma. The drastic reduction in the VAS score for leg pain following surgical decompression (from 9/10 to 1/10) further corroborates the efficacy of early intervention in preventing permanent deficits. In addition to pain scores, the ODI demonstrated a significant improvement in functional capacity, transitioning from moderate disability (40%) to minimal disability (10%). These findings emphasize the positive impact of timely interventions on pain relief and functional recovery. An inverse relationship was observed between the time elapsed since symptom onset and clinical outcomes, with earlier interventions associated with better results.^(25)^

## CONCLUSION

This case highlights the importance of recognizing perineural hematoma as a possible complication of lumbar injections, even in patients without traditional bleeding risk factors. Clinicians must remain vigilant for the possibility of vascular injury and the effects of increased epidural pressure during these procedures. The inclusion of the Visual Analog Scale and Oswestry Disability Index scores as metrics for assessing pain progression emphasizes the need for continuous monitoring. Early diagnosis and timely surgical intervention are crucial to prevent irreversible neurological damage.
